# Significance of Brain Glucose Hypometabolism, Altered Insulin Signal Transduction, and Insulin Resistance in Several Neurological Diseases

**DOI:** 10.3389/fendo.2022.873301

**Published:** 2022-05-09

**Authors:** Enrique Blázquez, Verónica Hurtado-Carneiro, Yannick LeBaut-Ayuso, Esther Velázquez, Luis García-García, Francisca Gómez-Oliver, Juan Miguel Ruiz-Albusac, Jesús Ávila, Miguel Ángel Pozo

**Affiliations:** ^1^ Department of Biochemistry and Molecular Biology, Faculty of Medicine, Complutense University, Madrid, Spain; ^2^ Department of Physiology, Faculty of Medicine, Complutense University, Madrid, Spain; ^3^ Pluridisciplinary Institute, Complutense University, IdISSC, Madrid, Spain; ^4^ Department of Pharmacology, Pharmacognosy and Botany, Faculty of Pharmacy, Complutense University, Madrid, Spain; ^5^ Center of Molecular Biology “Severo Ochoa”, CSIC-UAM, Madrid, Spain

**Keywords:** brain, glucose hypometabolism, altered insulin signaling, insulin resistance, neurological disorders

## Abstract

Several neurological diseases share pathological alterations, even though they differ in their etiology. Neuroinflammation, altered brain glucose metabolism, oxidative stress, mitochondrial dysfunction and amyloidosis are biological events found in those neurological disorders. Altered insulin-mediated signaling and brain glucose hypometabolism are characteristic signs observed in the brains of patients with certain neurological diseases, but also others such as type 2 diabetes mellitus and vascular diseases. Thus, significant reductions in insulin receptor autophosphorylation and Akt kinase activity, and increased GSK-3 activity and insulin resistance, have been reported in these neurological diseases as contributing to the decline in cognitive function. Supporting this relationship is the fact that nasal and hippocampal insulin administration has been found to improve cognitive function. Additionally, brain glucose hypometabolism precedes the unmistakable clinical manifestations of some of these diseases by years, which may become a useful early biomarker. Deficiencies in the major pathways of oxidative energy metabolism have been reported in patients with several of these neurological diseases, which supports the hypothesis of their metabolic background. This review remarks on the significance of insulin and brain glucose metabolism alterations as keystone common pathogenic substrates for certain neurological diseases, highlighting new potential targets.

## Introduction

**Figure 1:**
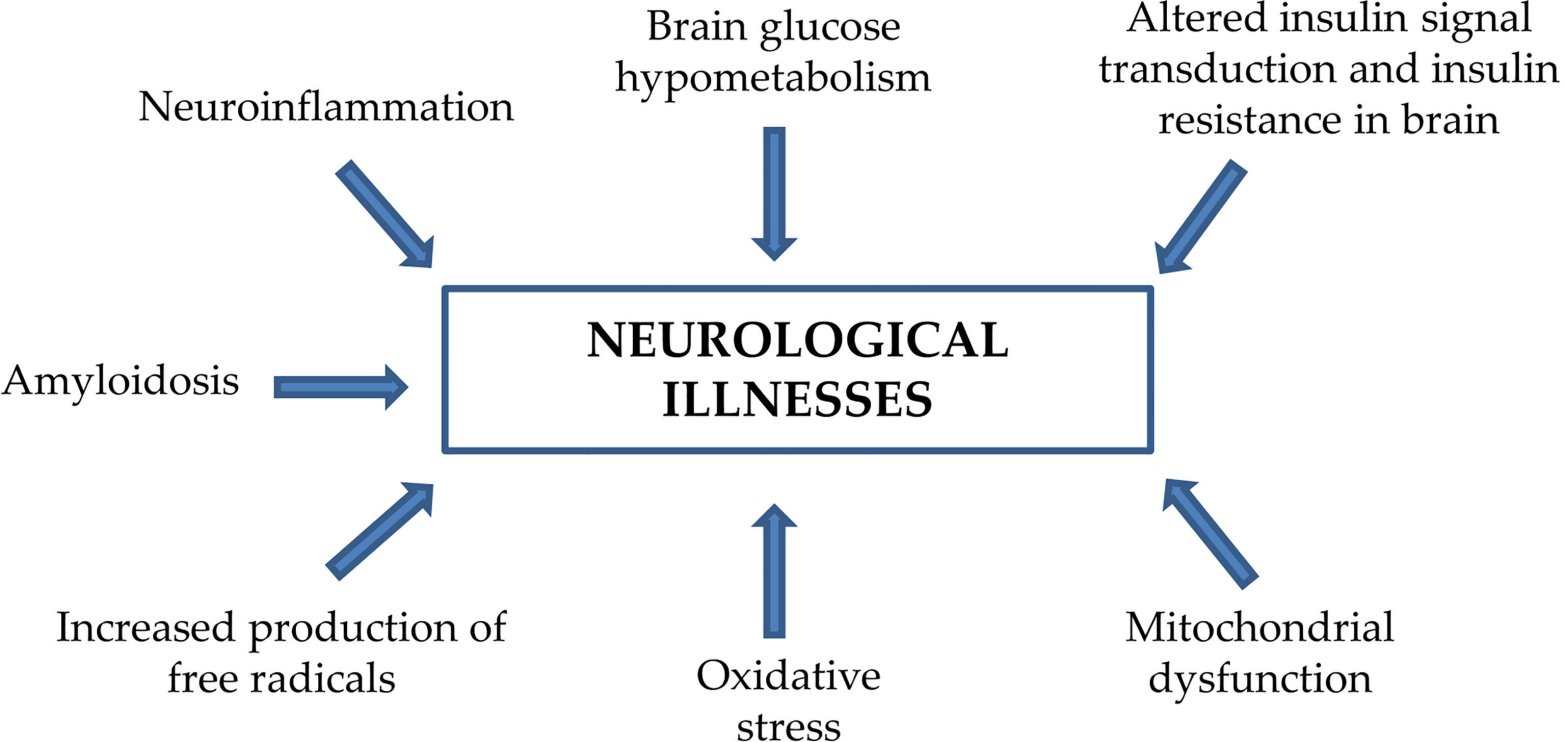
Expression of common pathogenic manifestations in the brain of patients with several neurological diseases.

Neurodegenerative diseases such as Alzheimer’s disease (AD), Parkinson’s disease (PD), Huntington’s disease (HD), and epilepsy-related disorders (EDs) (1) are characterized by central progressive alterations affecting many different brain structures. These neuropathologies and other chronic mental disorders such as schizophrenia (2) and major depressive disorder (MDD) have different etiologies but share common pathogenic manifestations such as neuroinflammation (3), brain glucose hypometabolism (4), oxidative stress (5), mitochondrial dysfunction (6), amyloidosis (7), insulin resistance (8, 9), and/or molecular alterations regarding insulin receptors and the insulin-induced signal transduction pathway (10) (**Figure 1**). In addition, these neurological diseases show high comorbidity with other pathologies such as type 2 diabetes mellitus (T2DM) and vascular diseases (11).

Neuroinflammation is present in the early stages of these diseases. The activation of microglia and astrocytes results in cytokine release (12), contributing to the initiation of the inflammatory process, which, in turn, may facilitate neural dysfunction and cell death (13). Brain injury, stroke, hypoxia, T2DM and vascular dysfunction are also considered risk factors that contribute to the development of glucose-metabolism disorders and induce oxidative stress, in addition to being primary pathways of the progression of the diseases, thus setting in motion a vicious circle (13). In addition, chronic inflammation potentiates resistance to insulin and insulin-like growth factor-1 (IGF-1) in the brain, as manifested in AD and PD (14). Furthermore, brain inflammatory processes cause neurotoxicity and hyperexcitability, which may facilitate epileptiform activity (15). A population-based study in PD patients, including matched controls, suggested a protective role for non-steroidal anti-inflammatory agents in PD (16).

Amyloid deposits are also common in AD, PD, and other neurodegenerative diseases (7, 17). Whereas deposits of islet amyloid polypeptide (IAPP) are observed in the islets of Langerhans in T2DM, amyloid β-peptide (Aβ) is the main component of the brain amyloid plaques in AD. These peptides are misfolded and self-assemble into oligomers and fibers that are able to form amyloid insoluble aggregates (18). The Aβ-42 peptide is known to induce neuronal toxicity, whereas IAPP is toxic in the pancreatic islet cells (19). These two proteins have a high sequence similarity, and the chaperone protein pathway preventing IAPP and Aβ aggregation might be common. It was suggested that the limited capacity of this shared chaperone protein is responsible for the development of AD and T2DM (20). Inflammatory responses are closely associated with the development of insulin resistance, and, under these conditions, the formation and deposition of amyloid plaques increase.

Studies in experimental animals showed that the peripheral or central administration of insulin by intranasal, intracerebroventricular (icv) or intrahippocampal routes has positive effects on memory and learning processes (21). The cognitive improvement could be related to increased hippocampal insulin receptor expression and/or to insulin-induced signaling transduction (22). In this context, memory loss due to a hippocampal ischemic lesion can be prevented by insulin administration (23). Additionally, the central administration of low doses of streptozotocin to adult rats induces central resistance by altering the binding of insulin to its receptor and blocking insulin’s action. Little evidence exists regarding the desensitization of the insulin receptor (IR); however, central insulin resistance has been shown to be related to cognitive and behavioral deficits (24).

Despite the specific molecular and cellular mechanisms, brain areas predominantly affected, different clinical presentation and therapeutical approaches used to their mainly symptomatologic treatment, these neurological illnesses also share common features.

## Relationships Between Cerebral Glucose Metabolism and Insulin Action

### Brain Glucose Metabolism

Glucose homeostasis requires hormonally and neurally mediated regulatory actions that contribute to the correct functioning of the brain and the peripheral tissues. Glucose is not only the main energy source for the maintenance of neural and non-neural cellular activity; it also acts as a signaling molecule. Therefore, glucoregulatory mechanisms are key to ensuring an appropriate glucose supply to meet the metabolic needs of the central nervous system as well as the peripheral tissues.

The antagonistic effects of the pancreatic hormones insulin and glucagon, the activity of the hypothalamic–pituitary–adrenal axis, and the components of the autonomic nervous system help to maintain blood glucose levels within a physiological range depending on the energy status of the organism. Alterations in physiological glycemic levels have deleterious consequences, increasing both morbidity and mortality rates. To prevent marked blood glucose oscillations, glucose sensors in several locations accurately sense the glucose concentrations in the extracellular space and set in motion regulatory mechanisms needed to maintain glucose homeostasis. Glucokinase (GK) has been shown to act as a glucose sensor in the hypothalamic neurons of both humans and rats (9, 25, 26), being involved in the regulation of energy homeostasis, feeding behavior, glucose metabolism, and the autonomic nervous system (27–29).

In humans under physiological situations, glucagon-like peptide-1 (GLP-1) was shown to significantly reduce glucose metabolism in a selective and temporal manner in the hypothalamus and the brainstem, areas involved in the control of food intake and glucose sensing (30). These observations suggest that reversible and brief glucose hypometabolism in these brain areas may induce important biological effects.

In the brain, virtually all glucose is oxidized to CO_2_ and H_2_O through glycolysis and the mitochondrial respiratory machinery. The relationship between O_2_ consumption and CO_2_ production in the brain is close to one, from which it follows that carbohydrates and glucose, in particular, are the exclusive substrates of oxidative metabolism in the brain (31). Changes in brain glucose metabolism detected using several technical approaches have been reported in AD patients. Currently, functional neuroimaging techniques such as positron emission tomography (PET) provide *in vivo* measurements of glucose metabolism with very high sensitivity. This imaging methodology allows us to identify functional changes in brain metabolic activity even before the appearance of clinical symptoms. At present, the use of ^18^F-FDG for PET imaging is approved in Europe and the USA for the study of glucose metabolism in certain cardiovascular, neurological, and oncological diseases (32).

**Figure 2:**
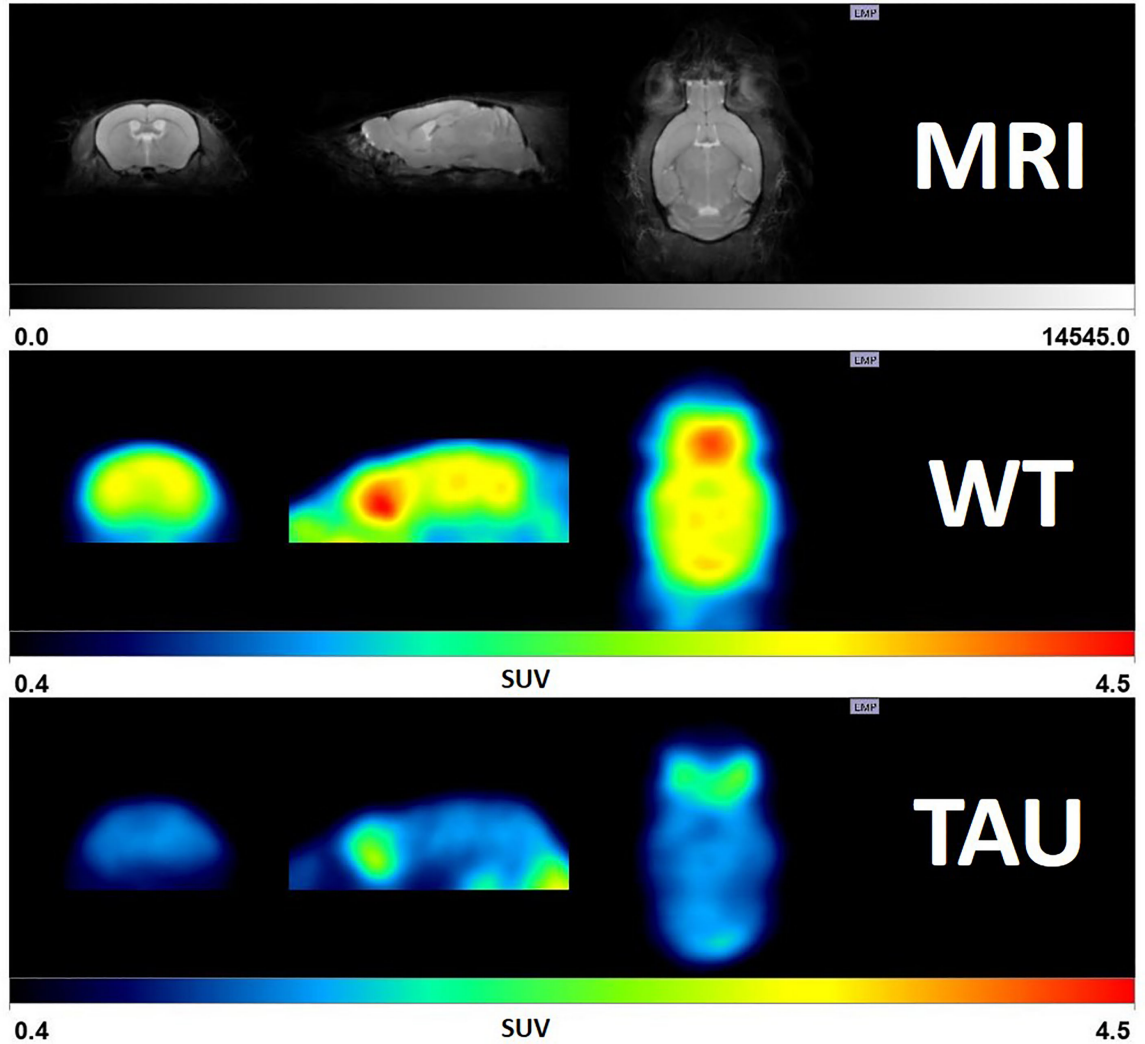
Glucose uptake in a transgenic model of tauophathy (TAUwlv transgenic mice) compared to control (wild type) animals at the age of 19 months, as detected by 18F-FDG PET neuroimaging. The upper row shows a MRI mouse brain template (coronal, sagittal and transversal views); the middle and the bottom rows show the PET image (normalized to SUV -standard uptake value-) corresponding to a representative wild-type and TAUvlw mouse, respectively.

In PET functional imaging, 2-deoxy-2-(^18^F) fluoro-D-glucose (^18^F-FDG) competes with glucose to bind to transporters localized in the cell membranes of neurons and astrocytes. The ^18^F-FDG intracellular concentration relates to the hexokinase activity and glycolysis of brain cells, and changes in glycolytic activity are associated with neurodegenerative diseases. Therefore, ^18^F-FDG-PET provides quantitative tomographic images of the distribution of neuronal metabolism, allowing the determination of the changes in regional hypometabolism observed in several neurological diseases. Thus, PET neuroimaging is a valuable tool for the early diagnosis of neurodegenerative diseases, which are characterized by marked alterations in brain glucose metabolism (**Table 1**). Regional brain glucose hypometabolism measured by ^18^F-FDG-PET imaging by itself indicates a reduction in cellular glucose utilization but this cannot be directly attributed to a particular type of cell (neuronal or non-neuronal) or whether is related to functional deafferentation or to cellular loss. Despite this caveat, considering that the affected areas have been well characterized, by means of other complementary techniques, by glial reactivity and neuronal dysfunction or death, it is generally accepted that brain glucose hypometabolism is mostly an overall reflection of neuronal impairment or death. Regional brain hypometabolism in neurodegenerative diseases is well-described in animal models of AD, in which hypometabolism is found in the hippocampus and temporo-parietal cortex. Thus, ^18^F-FDG-PET imaging in patients revealed that glucose metabolic reductions in the parieto-temporal, frontal and posterior cingulate cortices were a hallmark of AD (33). This focal alteration in metabolism can also be observed in brain areas affected by stroke and in epileptic foci. Likewise, brain glucose hypometabolism is typical in the striatum in PD models. In psychiatric pathologies such as major depression, robust hypometabolism is generalized. Furthermore, ^18^F-FDG-PET imaging can be performed in experimental models of disease. Thus, in a transgenic mouse model of tauopathy (34) as well as in GSK-3β-overexpressing mice (35), brain glucose hypometabolism is reflected by a reduction in ^18^F-FDG (**Figure 2**).

**Table 1:** Some aspects of brain glucose metabolism in health and disease.

	Glucose transporters	Glucose phosphorylating enzymes	Pathways of oxidative/energy metabolism	References
**In Health**	GLUT-1 and GLUT-3, the most abundant GLUT-2, GLUT-4, and GLUT-8, lower contents in selective areas	Hexokinase I is the most abundant	Normal functioning of glycolysis, Krebs cycle, oxidative phosphorylation, pentose route and thiamine metabolism	(10, 25–34)
		Glucokinase or hexokinase IV is considered a cerebral glucose sensor in the control of food intake		
**In Disease**	Glucose-metabolism dysfunction increases the risk of cognitive impairment; reduced GLUT-1 and GLUT-3 expression in several diseases	Characterizing brain glucokinase mutations related to nosological entities, as happens in liver and pancreatic beta cells, should be of interest.	Mitochondrial dysfunction and oxidative stress; alterations in Krebs cycle, oxidative phosphorylation and thiamine metabolism	(34–40)

### Brain Insulin Actions

Insulin action inducing glucose uptake in the CNS differs from that inducing that in the peripheral tissues due to the brain’s lower content of GLUT-4, which is the only insulin-sensitive glucose transporter. However, GLUT-1 and GLUT-3’s presence and action are more relevant in the brain than in peripheral tissues. This selective tissue distribution of glucose transporters is necessary for the metabolic activities occurring in physiological situations and might explain the alterations observed in pathophysiological conditions (34, 35)

Brain glucose uptake was traditionally considered insulin-independent (41, 42). It is currently known that insulin acts in concert with IGF-1 on astrocytes controlling brain glucose metabolism (43, 44). Thus, insulin and IGF-1 synergistically stimulate a mitogen-activated protein kinase/protein kinase D (MAPK/PKD) pathway, resulting in the translocation of GLUT-1 to the cell membrane through multiple protein–protein interactions.

The ablation of insulin receptors in astrocytes reduces the glucose-induced activation of hypothalamic proopiomelanocortin (POMC) neurons. Accordingly, astrocytic insulin signaling co-regulates hypothalamic glucose sensing and systemic glucose metabolism. Thus, insulin signaling in hypothalamic astrocytes co-controls CNS glucose sensing and systemic glucose metabolism *via* the regulation of glucose uptake across the blood–brain barrier (BBB) (45). In this context, the role of hypothalamic GK as a glucose sensor involved in the control of satiety and body weight is noteworthy. At this point, it is important to know the functional interrelations between GK and GLUT-1 in POMC neurons.

Despite the different physiological consequences, the molecular events through which insulin acts on the brain are similar to those in the periphery (**Table 2**).

**Table 2:** Some aspects of physiological and pathophysiological metabolism of insulin in the brain.

	Insulin signaling	Insulin actions	References
**In Health**	-Brain insulin receptors have similar properties to those described in peripheral tissues. -Acting through IR/IRS-1/PI3K/Akt/mTOR and MAPK pathways	- On energy expenditure, feeding behavior, glucose homeostasis and reproduction. - Neuroprotective effects - Neuromodulatory effects on cognition, learning and memory	(10, 43–50)
	**Alterations of insulin receptor signaling**	**Insulin resistance**	
**In Disease**	-Reduced brain insulin receptor, PI3K/Akt pathway, and overactivation in GSK-3β in AD, T2DM, and schizophrenia - Alterations in Akt activity in HD	- Present in epilepsy, PD, AD, T2DM, and schizophrenia - Risk factor for cognitive impairment; insulin improves it - Affects hippocampal plasticity, APP metabolism, and brain inflammatory reactions, and increases tau protein concentrations	(51–59)

Insulin’s actions on the brain contribute to regulating energy expenditure, glucose homeostasis, feeding behaviors, reproduction, cell proliferation and differentiation, neuroprotection, neuromodulation, and learning and memory (10). When referring to insulin-induced proliferative effects, the interaction of insulin and IGF-1 at the receptor molecular level must be considered. Insulin can act on IGF-1 receptors at high concentrations. Likewise, IGF-1 can also interact with the insulin receptor. In addition, there are hybrid receptors in which one of the α-subunits binds to insulin, while the other binds to IGF-1. These findings, increasing the plasticity of both molecules in growth and metabolic activities, may be relevant under physiological and pathological situations.

Insulin is also a potent neuroprotective agent, mainly inhibiting apoptosis, beta amyloid toxicity, oxidative stress and ischemia (46–50). Moreover, insulin facilitates learning and memory by modulating hippocampal synaptic plasticity, which is importantly regulated by glutamatergic neurotransmission, which mediates long-term depression (LTD) by reducing AMPA receptors in the postsynaptic membrane while stimulating long-term potentiation (LTP). Glutamate also upregulates GABA receptors in the postsynaptic and dendritic membranes of the neurons (60–62). Impaired memory ability and reduced hippocampal synaptic plasticity were shown to be restored by insulin treatment in an experimental model of diabetes mellitus. Additionally, it was reported that IGF-1 augments hippocampal synaptic transmission by a mechanism involving AMPA receptors and PI3K activity (63).

Insulin also affects APP metabolism, activating α-secretases and regulating Aβ levels by promoting Aβ transport to the neuronal gap. Insulin-degrading enzyme (IDE) is responsible for degrading not only insulin but also Aβ, having a higher affinity for the hormone than for Aβ. Then, insulin can prevent the formation of Aβ fibrils, stimulating the internalization of Aβ oligomers and, therefore, inhibiting their binding to neurons, protecting the synapses from Aβ oligomers (64). In states of insulin resistance, the protective role of insulin regarding Aβ accumulation is diminished, and, in turn, Aβ deposits downregulate the action of insulin. Consequently, Aβ peptides inhibit the binding of insulin to its receptors, altering insulin-induced signaling pathways (65, 66). It was shown that tau protein phosphorylation increased significantly in an animal model of insulin resistance induced by fructose; in AD transgenic mice, insulin resistance induced by diet facilitated brain Aβ formation.

## Alterations in Glucose and Insulin Metabolism in Neurological Diseases

Insulin alterations and changes in glucose metabolism (67) were suggested to be risk factors for developing certain neurological diseases (**Tables 1**, **2**). Thus, individuals with elevated circulating blood glucose concentrations are not only at a higher risk of developing dementia (20, 68, 69) but also progress more rapidly from mild cognitive impairment (MCI) to AD. These findings suggest that abnormal glucose metabolism may play a role in the pathogenesis underlying AD. Hypoglycemia is relatively frequent in diabetic patients treated with either insulin or oral hypoglycemic agents. Likewise, hypoglycemia is manifested in nondiabetic patients with insulinoma, severe liver illness, some endocrine diseases and alcoholism. A chronic state of cerebral glucose hypometabolism was recently identified in people with several neurodegenerative diseases such as partial epilepsy, PD, AD, schizophrenia, and HD, and in MDD patients (13). Brain glucose hypometabolism appears early in the preclinical stages of these diseases and contributes to their pathogenic manifestations. Studies with ^18^F-FDG-PET imaging showed a significant decrease in brain glucose metabolism in MCI patients in the earlier stages of AD (70) as well as in other neurodegenerative diseases such as PD (71). Altogether, hypometabolism may be an important biomarker of the pathogenic course, preceding the clinical manifestations of the disease. The main scientific societies recommend the use of ^18^F-FDG PET in patients with AD in the prodromal phase or MCI (72)

Because brain glucose hypometabolism precedes the first clinical manifestations by years, it is not likely that significant neuronal loss accounts for the lower ^18^F-FDG-PET signal (4). Furthermore, oxidative stress related to inflammation, misfolded protein toxicity, mitochondrial dysfunction and impaired glucose metabolism contributes to neuronal death and neural dysfunction (2, 73).

### Brain Glucose Metabolism and Neuroinflammation in Alzheimer Disease and Type 2 Diabetes Mellitus Patients

AD is a neurological disorder that causes significant memory loss and progressive dementia, accompanied by the presence of amyloid plaques, neurofibrillary tangles and amyloid angiopathy, as well as the widespread loss of neurons and synapses (74). More than 40 million people worldwide suffer from AD. The prevalence is increasing rapidly, being expected to exceed 130 million patients by 2050 (51). Impaired insulin secretion and/or insulin resistance were estimated to affect 425 million patients worldwide in 2017, with 90% of the patients having a diagnosis of T2DM (75).

Preclinical studies reported that the induction of acute hyperglycemia in a mouse model of AD, using glucose clamps and *in vivo* microdialysis techniques, increased Aβ and lactate production in the hippocampal interstitial fluid (ISF), considered biomarkers of altered neural activity. These effects were exacerbated in aged AD mice with advanced Aβ pathology. Furthermore, and based on the altered ISF Aβ levels and neuronal activity observed after the pharmacological manipulation of ATP-sensitive potassium (K^+^-ATP) channels in the hippocampus, they suggested the involvement of these channels in mediating the response to hyperglycemia. Therefore, K^+^-ATP activation might mediate the response of hippocampal neurons to hyperglycemia by coupling metabolism with neuronal activity and ISF Aβ levels (76). Likewise, *in vitro* studies showed that Aβ1-42 incubated with hippocampal slices produced a significant decrease in glucose uptake (77), which was related to impaired glycolysis but not to altered mitochondrial function (70, 78).

Insulin resistance is a risk factor for the development of AD, being a common finding in AD patients independent of T2DM. Furthermore, peripheral insulin resistance might also be accompanied by central insulin resistance, IGF-1 resistance, and IRS-1 and IRS-2 dysfunction, presumably as a consequence of Aβ oligomers further contributing to cognitive decline (79). It was proposed that peripheral and central insulin resistance crosstalk through a “liver–brain axis”, promoting cognitive dysfunction in T2DM (80).

Clear-cut experimental evidence shows that inflammatory responses are closely associated with the development of insulin resistance in obesity and T2DM (81), increasing the risk of AD. Accordingly, in the cerebrospinal fluid (CSF) of patients with AD, high concentrations of interleukin 6 (IL-6) have been reported (82). Studies on experimental animals suggest that inflammation interacts with the processing and deposition of β-amyloid peptide (83). Increased levels of inflammatory cytokines alter hippocampal synaptic plasticity and the components of spatial learning (84). Chronic inflammation may contribute to the development of insulin resistance and T2DM, as well as the association of both AD and T2DM (85). Additionally, augmented brain TNFα and Aβ contents in obese hyperinsulinemic patients facilitate the formation of amyloid plaques (86).

Alterations in some insulin signaling pathways such as PI3K/Akt and GSK-3 are present in central inflammation and insulin resistance (51). The PI3K pathway inhibits the formation of IL-12 by dendritic cells, whereas GSK-3 is a kinase involved in tau hyperphosphorylation and the modulation of Aβ metabolism (87). Some interactions occur between this protein and the insulin signaling pathway. IR activation phosphorylates and inhibits GSK-3β (52). In AD and T2DM, GSK-3β activity is increased, phosphorylating the IR and IRS-1 (53). In addition, the inhibition of GSK-3 stimulates the production of anti-inflammatory cytokines such as IL-10, and decreases proinflammatory cytokines such as IL-1β, IL-6 and IFN-ϒ in response to Toll-like receptors (88). These findings have been observed in AD patients and in animal models. Accordingly, the PI3K/Akt/GSK-3 pathway may play a relevant role by mediating the inhibitory effect of insulin on inflammation.

^18^F-FDG-PET imaging has revealed reduced glucose uptake in several brain areas in AD patients (89, 90). This brain glucose hypometabolism is present in both nondiabetic and diabetic patients with AD. However, it is important to remember that glucose metabolism dysfunction in diabetic patients increases the risk of cognitive impairment (91). In transgenic mice overexpressing the tau protein, marked hippocampal glucose hypometabolism and a significant beneficial effect of insulin improving cognitive function were reported (34).

### Brain Glucose Metabolism in Parkinson’s Disease: Relationship Between Diabetes Mellitus and Parkinson’s Disease

Parkinson’s disease (PD) is a neurodegenerative disorder caused by a progressive deterioration of the dopaminergic neurons of the substantia nigra in the midbrain (92). PD patients exhibit widespread cortical hypoperfusion and reduced brain glucose metabolism (71). As in AD patients, these metabolic abnormalities are expressed in the initial stages of the disease, suggesting that they might be important for an early diagnosis of the disease or for initiating potential treatment.

Several pieces of epidemiological and molecular evidence show that insulin alterations such as DM are related to PD (93). The high density of insulin receptors in the dopaminergic neurons of the ventral tegmentum and substantia nigra suggests that those neurons might be targets for the action of insulin (54, 94–97). In addition, insulin receptors’ mRNA expression and immunoreactivity in the neurons of the substantia nigra are reduced in PD patients (98), and dysfunctional insulin-mediated signaling occurs before the death of dopaminergic neurons. Therefore, it seems that a relationship between insulin dysregulation and PD exists.

Approximately half of the patients with a diagnosis of PD are either glucose-intolerant and/or diabetics (99). Data obtained from postmortem studies, experimental animal models and cell cultures support the idea that the neuroinflammatory processes related to microglial activation, astrogliosis, and lymphocyte infiltration are involved in the loss of neurons in PD (100–102). Considering the role of neuroinflammation in insulin resistance and the role of the components involved in insulin signaling transduction mediating inflammatory processes, it could be deduced that neuroinflammation may facilitate the crosstalk between insulin dysfunction and PD. In this context, the antidiabetic drug pioglitazone, acting through peroxisome proliferator-activated receptor gamma (PPAR-Υ) receptors, was interestingly shown to reduce neuroinflammation, suggesting that it might protect dopaminergic neurons in PD (103).

Insulin dysfunction has also been related to the progression of PD. It was shown that dopamine synthesis in the synaptosomes of the striatum in diabetic rats was significantly lower than that in the controls, and insulin resistance reduced the release and clearance of dopamine by dopaminergic neurons (104).

Insulin has regulatory effects on the expression and activity of tyrosine hydroxylase, the limiting enzyme responsible for dopamine synthesis (105). Thus, tyrosine hydroxylase activity and dopamine concentrations are reduced in the PD brain; reduced tyrosine hydroxylase mRNA expression was reported in the dopaminergic neurons of diabetic rats (106). In addition, insulin has been shown to increase the expression of the dopamine transporter and to promote presynaptic dopamine reuptake in the tegmentum ventral and substantia nigra (107, 108).

^18^F-FDG-PET imaging studies showed that glucose metabolism is elevated in the putamen, an increase that is usually accompanied by low ^18^F-DOPA uptake. Other brain regions of the associative cortex show a decline in glucose metabolism that increases as the disease progresses. Importantly, progressive cortical hypometabolism in temporo-parietal and occipital regions marks the transition from normal cognition to dementia (109).

### Alterations in Cerebral Glucose Metabolism and Insulin in Patients With Huntington’s Disease

Huntington’s disease (HD) is a neurodegenerative genetic disorder characterized by the abnormal formation of polyglutamine aggregates, known as huntingtin, which progressively results in neuron dysfunction and tissue loss in the striatum and cerebral cortex. The clinical manifestations of HD include disordered movements, cognitive decline, and psychiatric symptoms (55). Additionally, HD is not only associated with altered insulin metabolism and diabetes mellitus, but also has a neuroinflammatory component that plays a relevant role in the development and progression of the disease (110). Insulin-sensitizer drugs, such as thiazolidinediones, and anti-inflammatory drugs, such as inhibitors of cyclooxygenase (COX-2), have been shown to have protective effects in experimental models of HD (111, 112).

The incidence of DM is higher in HD patients (113), who have abnormal glucose tolerance (114), likely as a consequence of reduced β-cell mass, insulin content, and β-cell replication, and altered exocytosis (115). The glibenclamide treatment of diabetes in a transgenic mice model of HD showed beneficial effects, suggesting a stimulatory action on insulin exocytosis (116).

Insulin plays a key role in regulating genes involved in the pathogenesis of HD, and insulin dysregulation affects the neuropathology of HD (117). The activity of the protein kinase Akt, one of the main components of the insulin signaling pathway, is altered in HD. The activation of Akt in the insulin signaling cascade inhibits cell death in primary cell cultures of the striatum. Results from postmortem studies with HD samples demonstrate that caspase-3 degrades Akt, abolishing its effect on cell survival (55).

Another mechanism underlying the effect of insulin on the pathogenesis of HD is the ability of this hormone to promote the clearance of huntingtin aggregates. Although neurons have a certain ability to clear these protein aggregates, the continuous production of the mutant protein, as occurs in HD, compromises this ability. We suggest that a vicious circle between the pathogenesis of HD and insulin-related alterations might develop, contributing to the progression of the disease. In addition, metformin is considered by some authors as therapeutically useful for neurological diseases such as HD (118).

^18^F-FDG-PET imaging studies in HD revealed an early and marked reduction in glucose metabolism in the caudate and putamen nucleus that, in further advanced stages, extends to other brain areas such as the thalamus and brain cortex (119).

### Epilepsy and Brain Glucose Hypometabolism

Epilepsy is a heterogeneous neurological chronic disease characterized by recurrent spontaneous seizures. It is one of the most important neurologic syndromes, with a high prevalence, affecting around 0.5–2% of the world’s population. Among the different forms of this disease, temporal lobe epilepsy (TLE) is the most prevalent in adults. The development of epilepsy is associated with a wide spectrum of neuronal alterations, including neuroinflammation, neurochemical imbalances, synaptic modifications in specific brain areas, and metabolic activity disturbances. ^18^F-FDG-PET imaging techniques, commonly used in TLE patients, have been demonstrated to be highly sensitive, allowing for the localization of the epileptic focus. During the seizure or ictal phase of the disease, the brain metabolism and cerebral blood flow increase in the epileptic focus. By contrast, after the seizure, during the interictal phase, the epileptogenic zone is characterized by pronounced glucose hypometabolism (120).

Under physiological conditions, brain metabolism is mainly fueled by glucose through both aerobic and anaerobic pathways (121). It was suggested that altered glucose metabolism is probably a key initiating factor promoting epileptogenesis. Energy deficiency could underlie the brain hypometabolism featured by ^18^F-FDG-PET imaging in patients and in animal models of epilepsy (122, 123).

In epilepsy, during seizure, brain glucose hypermetabolism occurs (124–126), and the metabolic rate of glucose and oxygen consumption increases. It was suggested that the aerobic pathway is unable to supply the amount of energy required to deal with the demands imposed by the seizure. The energy necessary to sustain a neuronal-hyperactivity-induced seizure might be obtained *via* increased blood glucose uptake and/or increased glycolytic astrocyte activity coupled to the lactate shuttle (121). The inhibition of lactate dehydrogenase (LDH) reduces hippocampal lactate, eliciting neuronal inhibition and reducing seizures and epileptiform activity (127). It has long been known that a ketogenic diet is a nonpharmacological alternative treatment for children with refractory epilepsy. Less is known about its effectiveness in adult patients. Nevertheless, the mechanisms underlying the antiepileptogenic effect of ketogenic diets are yet to be fully unveiled. Furthermore, ketogenic diets seem to act through many distinct and unrelated mechanisms that ultimately converge in an overall antiepileptogenic effect. Nevertheless, it seems that ketogenic diets, by leading to the generation of ketone bodies as alternative fuel, reduce hippocampal lactate by weakening the astrocyte–neuron lactate shuttle. Thus, both LDH inhibition and ketogenic diets seem to converge in at least one common mechanism related to their antiepileptogenic properties.

Among the various animal models of epilepsy that have been developed, the most frequently used are those that induce partial seizures through kindling or status epilepticus (SE). Kindling is the procedure through which the progressive development of seizures is achieved in response to an initial subconvulsive electric or chemical stimulus applied repeatedly and intermittently (128).

In addition to its clinical application, ^18^F-FDG-PET imaging has been used to characterize several animal models of epilepsy, such as the lithium–pilocarpine status epilepticus and amygdala electrical kindling models (12, 129). For studies in those models as well as in other animal models of epilepsy, ^18^F-FDG-PET imaging reveals a significant reduction in cerebral glucose metabolism. In the silent stage of the pentylenetetrazol (PTZ) kindling model (130), this glucose hypometabolism was considered to be a reliable and predictive index for the epilepsy outcome, clearly correlating hypometabolism imaging in the entorhinal cortex and epileptogenesis with spontaneous seizures (**Figure 2**). This finding suggests that a reduction in brain glucose metabolism is effectively associated with convulsive seizures. Although the mechanisms underpinning brain glucose hypometabolism in epilepsy are not yet fully understood, it was suggested that epilepsy-induced brain hypometabolism could be due to neural loss (131), the alteration of cerebral blood flow (132), or the deafferentation of epileptic neurons (133).

Altered insulin signaling in the brains of genetic-absence epilepsy rats from Strasbourg was found (134), suggesting a role for this hormone in this disorder and a possible relationship of it with brain glucose alterations.

Even though the mechanisms underlying brain glucose hypometabolism in epilepsy and their potential contributions to the neuropathology of epilepsy are still unknown, it is reasonable to suggest the involvement of multiple and likely concurrent processes such as neuronal excitability dysfunction, neuroinflammation and microglial activation, disturbed brain metabolism, neuronal death, and/or disruption of cerebral blood flow.

### Alterations of Cerebral Glucose Metabolism and of Insulin Action in Patients With Schizophrenia

Schizophrenia is a psychiatric disorder characterized by abnormal behavior and psychotic symptoms such as hallucinations, false beliefs, confused thinking, reduced social engagement, and emotional expression. At the neurochemical level, these patients have alterations in dopaminergic and glutamatergic neurotransmission. Although the mechanisms by which they occur are unknown, progressive inflammation and neurodegeneration have been suggested as the potential main mechanisms contributing to this disease (135, 136). Similar to the other neuropathologies discussed in this review, alterations in the secretion of and sensitivity to insulin also occur in schizophrenic patients (56, 137). Schizophrenic individuals have elevated circulating insulin levels, which corresponds to a state of insulin resistance, and are more susceptible to suffering from T2DM (138), and they have altered tolerance to glucose overload. Additionally, schizophrenic patients with normal body weights and corporal mass indices have anomalous tolerance to glucose, and they have abnormalities in lipid and energy metabolism (139).

It is known that the signal transduction mediated by the insulin receptor located in the prefrontal dorsolateral cortex is reduced, as is the autophosphorylation of the insulin receptor (138). Clozapine administration in animal models of insulin resistance reduced the signaling deficiency for the insulin receptor (138).

Patients with schizophrenia were found to have frontal hypometabolism in ^18^F-FDG-PET studies, and this was found to be strongly associated with cognitive deficits (140).

### Alterations in Cerebral Insulin Action in Patients With Major Depressive Disorder

Robust evidence supports the idea that DM is associated with changes in mood and anxiety (141). Many studies have established a marked relationship between depressive alterations and changes in insulin metabolism. The prevalence of depression in T2DM patients is three times greater than that in the general population (142). This is also observed in the pediatric population, with depression being two to three times more prevalent in diabetic children and adolescents than in their nondiabetic counterparts. Mood alterations during the postpartum period have also been related to the abrupt decrease in circulating insulin levels after delivery (143).

Insulin is known to have effects on serotonin neurotransmission, which plays an important role in behavior and mood disorders. Most of the antidepressant drugs act by increasing the synaptic concentrations of serotonin and other monoamines such as dopamine and norepinephrine (144). Insulin metabolism alters brain monoamine activity both in diabetic humans and in animal models of DM (11). The serotonin content in the hypothalamus and brain stem of streptozotocin-diabetic rats is reduced (145), and changes in the expression and function of brain dopamine and serotonin receptors were also observed in the brain cortices of such animals (139). Thus, although only the density of the 5HT2a receptor was increased, both 5HT2a and 5HT1a receptors showed a reduced sensitivity in response to their agonists, which was restored to the values found in the control animals after insulin treatment (139).

Depression has negative effects on glycemic control, being a high-risk factor for the development of DM (146). It was suggested that the persistent activation of the hypothalamus–pituitary–adrenal gland axis in depression may facilitate the occurrence of T2DM (147).

Depression is also associated with neuroinflammatory and neurodegenerative processes (148). Thus, the levels of proinflammatory cytokines are increased in the blood plasma, CSF, and samples of postmortem brains of patients with severe depression; interestingly, treatment with antidepressant drugs normalizes the elevated levels of cytokines (149). The administration of cytokines, such as IL-1β, induces anxiety behavior and depression, whereas treatment with anti-inflammatory agents, such as long-chain polyunsaturated fatty acids, produces antidepressant effects (150, 151).

One of the mechanisms described as responsible for the depressive state induced by inflammation is altered brain monoaminergic neurotransmission. Accordingly, the central administration of IL-1β increases the production of dopamine, norepinephrine and serotonin, which also occurs in diabetic brains (150).

As previously mentioned, insulin dysfunction plays a crucial role in neuroinflammatory processes. Thus, neuroinflammation might be a common phenomenon that connects the neurodegenerative and neuropsychiatric diseases with insulin dysfunction (152, 153).

Molecular, functional and structural changes have been detected in the brains of major-depressive-disorder patients, mainly by means of imaging techniques such as magnetic resonance imaging (MRI) and positron emission tomography (PET) (154). An anomalous default mode network, as revealed by resting-state functional MRI, is probably associated with aberrant metabolic and serotonergic functions. These findings indicate that further investigations are important to shed light on the serotonergic network system associated with the behavior and genetic variations in major depressive disorder.

## Impaired Glucose Tolerance and Resistance to Insulin Action: Common Features of Several Nosological Entities

Many studies support a relationship between pathological conditions characterized by insulin dysfunction, such as impaired glucose tolerance, insulin resistance, and DM, and AD (155).

Insulin resistance, understood as a target organ’s reduced sensitivity to insulin, results in hyperinsulinemia but not in high insulin concentrations in the CSF, where the insulin concentrations are reduced. The latter may be explained by reduced insulin clearance and/or low insulin uptake from the peripheral sources (57). Accordingly, the mRNA expression and concentrations of insulin and IGF-1, and their transducers are reduced in the brains of AD patients (156). Furthermore, the administration of those proteins ameliorates some of the typical neuroanatomical signs of the brains of AD patients such as atrophy and mass loss (157).

Insulin resistance is known to contribute to the development of cognitive dysfunction due to, at least in part, the loss of the neuroprotective effects attributed to insulin. Thus, in several rodent models, a lack of insulin leads to neurodegeneration (158); accordingly, nasal or intrahippocampal insulin administration has been shown to improve spatial memory ability and cognitive function (58, 59). Similarly, pioglitazone, an insulin sensitizer, ameliorates the impairment in learning and memory functions. These findings support a possible neuroprotective role for insulin in other brain-damage conditions such as ischemia, apoptosis, oxidative stress and Aβ toxicity (51). The mechanisms underpinning the effects of insulin resistance on cognitive impairment include those affecting hippocampal plasticity, APP metabolism, increased tau protein concentrations, and neuroinflammation (57).

Altered cognitive function seems to be more frequent in patients with metabolic syndrome and those with central obesity (159, 160) than in the general population. Similarly, insulin resistance promotes the development of MCI and AD (161).

Impaired insulin transduction with reduced insulin and IGF-1 receptors (IR and IGF-1R, respectively); insulin and IGF-1 mRNA expression and protein concentrations; and downstream signaling elements, such as insulin receptor substrates 1 and 2 (IRS-1 and IRS-2) and phosphatidylinositol 3-kinase (PI3K), were observed in the brains of AD patients (156). Thus, AD is being associated with hypoactive IR/IRS-1/PI3K insulin signaling, even more noticeable with hypoactivity of the IGF-1R/IRS-2/PI3K signaling pathway (80, 162, 163).

Senile plaques composed of Aβ-peptide extracellular aggregates are a typical neuropathological feature of AD (164, 165) that also have significant effects on insulin signaling. Thus, Aβ-peptides were shown to inhibit the binding of insulin to its receptors, to reduce receptor autophosphorylation, to induce its downregulation by internalization and removal, and to impair insulin-induced signaling pathways (166, 167). Altogether, Aβ peptides contribute to the development of central insulin resistance; accordingly, the neuroprotective effects of insulin might be reduced in the AD brain as a consequence of both insulin expression and function being downregulated (168). Furthermore, the relationship between Aβ-peptides and insulin is reciprocal, as insulin induces the release of intracellular synaptic Aβ-peptide (48) and regulates the expression of insulin-degrading enzyme (IDE), a protease involved in the clearance of Aβ (169). Altogether, the reduced neuroprotective actions of insulin in AD and T2DM patients contribute to an increase in the brain´s vulnerability to neurodegeneration.

Intraneuronal neurofibrillary tangles (NFTs) (164, 165), mainly composed of hyperphosphorylated tau protein, are also a feature of the AD brain (170). Additionally, NFTs not only play an important role in AD, but their concentrations in the CSF are elevated in MCI patients with insulin resistance. However, the cerebellum is neither affected by reduced glucose hypometabolism nor devoid of NFTs (171).

The neuroprotective effects of insulin also seem to extend to anti-inflammatory-related mechanisms. Inflammatory factors such as IL-1, IL-6, and tumor necrosis factor-α (TNF-α) are augmented in the brains of AD patients (81), indicating the presence of a nonspecific immune inflammatory reaction in the early phase of brain plaque formation. Microglial cells contribute to the brain´s cellular immune reaction, playing a neuroprotective role *via* the phagocytosis of pathogenic microorganisms and dangerous particles (172). A lack of insulin-mediated actions in insulin resistance potentiates the microglia- and astrocyte-mediated inflammatory response in AD pathology. Thus, insulin resistance increases plasma insulin reactivity, reduces insulin sensitivity, and activates proinflammatory cytokines in the brain such as C-reactive protein and IL-6 (173). Augmented levels of proinflammatory cytokines can alter spatial learning related to hippocampal synaptic plasticity (84).

Glucose hypometabolism, likely related to insulin dysfunction, includes altered insulin transduction signaling, conditioned by different risk factors. In addition, glucose intolerance is observed in T2DM, schizophrenia, and HD and in more than 50% of PD patients (**Table 3**). In HD patients, both insulin secretion and its metabolism are altered (174). Furthermore, insulin was shown to play an important role in the control of the genes involved in HD etiopathology (55), inducing the expression of dopamine transporters and increasing dopamine clearance (117, 175).

**Table 3:** Alterations of glucose metabolism and of insulin action in brain of several neurological diseases and Type 2 Diabetes Mellitus.

DISEASES	GLUCOSE HYPOMETABOLISM	NEURO- INFLAMMATION	GLUCOSE INTOLERANCE	INSULIN EFFECTS	INSULIN RESISTANCE	ALTERATIONS OF INSULIN SIGNAL TRANDUCTION	REFERENCES
EPILEPSY	✓	✓	✓	Although Diabetes is an established risk factor for acquired epilepsy, there is a lack of reports showing how neural hiperactivity could influence brain insulin signaling	✓	Neuroprotective levels of IGF-1 exacerbate epileptogenesis after brain injury. These effects of IGF-1 were mediated by Akt-mTOR-signaling, which are also transducers of insulin action. However alterations in the insulin signaling of genetic absence of epilepsy in rats from Strasbourg has been found	(120, 130, 134)
PARKINSON	✓	✓	>50%	**↑** Expression of Dopamine transporter	✓	Alterations of insulin signal transduction	(54, 71, 98, 99, 104–109)
				**↑** Dopamine clearance			
ALZHEIMER	✓	✓	✓	Nasal insulin administration improved learning and memory	✓	Reduced brain insulin receptor/PI3K/Akt pathway and overactivation of GSK-3β	(52, 53, 81–85, 88–91)
SCHIZOPHRENIA	✓	✓	✓	Alteration of secretion and sensitivity to insulin	✓	Reduced brain insulin receptor/PI3K/Akt pathway and overactivation of GSK-3β	(56, 135–140)
HUNTINGTON	✓	✓	✓	Alteration of insulin secretion	✓	Alteration of Akt activity	(55, 110–118)
				↑ expression HD genes			
MAJOR DEPRESIVE DISORDERS	✓	✓	✓	Changes of cerebral monoamine activities, modifications of serotonin receptors	✓	-	(141–148, 150–154)
T2DM	✓	✓	✓	Metabolic parameters, memory and learning improved	✓	Reduced brain insulin receptor/PI3K/Akt pathway and overactivation of GSK-3β	(34, 51–53, 75, 80–91)

✓, means that is present. ↑, is increased; – means it is absent.

In this context, the nasal administration of insulin was shown to improve not only the metabolic parameters but also memory and learning in PD, AD, and T2DM patients. In HD patients, a significant reduction in IR and Akt kinase autophosphorylation as well as increased GSK-3β activity was found. Therefore, central insulin resistance may be due, at least in part, to a slow pathological progression of nosological entities, such as epilepsy, AD, T2DM and schizophrenia. In turn, central insulin resistance might facilitate the deterioration in hippocampal and cortical neurons with the progression of the illness, from its beginning, during its expansion, to its obvious clinical manifestations 15–20 years later (89). Further studies are needed to unveil to what extent this hypothesis might be valid, as well as the possibility that some neurodegenerative diseases such as AD or PD may be the consequence of a slow deteriorative process caused by viral, bacterial or fungal infections, or by the propagation of prion-like misfolded proteins (176–179).

## Significance of Other Biomolecules Besides Insulin That May Affect Brain Glucose Hypometabolism and Central Insulin Resistance

Based upon the large amount of information gathered in the last few decades regarding the central actions and effects of other hormones and regulatory peptides, we considered it necessary to mention some of the most relevant (**Table 4**). Thus, included in this group are the adipokines, molecules that regulate several metabolic actions, energy expenditure, insulin sensitivity and neuroendocrine function (190, 191).

**Table 4:** Roles of other molecules in addition to insulin that may modulate brain glucose metabolism and central insulin signal transduction.

Molecular candidates that alter brain glucose metabolism and central insulin actions	Biological effects	References
**Endocannabinoid System**	- Constituted by CB1 and CB2 receptors activated by endocannabinoid ligands - Blocking of CB1 receptors improves insulin sensitivity - Cannabinoid CB2 receptors agonists have neuroprotective and anti-inflammatory effects, stimulate brain glucose uptake, and reduce brain Aβ-induced cytokine release	(128, 180–182)
**Adiponectin**	↑Brain glucose metabolism, fatty oxidation, and brain insulin sensitivity	(183, 184)
	↓Adiponectin levels, contributing to the severity of T2DM	
	↑Adiponectin levels prevent the effect of high-fat-diet-induced obesity	
**Molecules from gut microbiome (such as lipopolysaccharides)**	Induce: -Neuroinflammation -Insulin resistance -Depression behavior	(180, 181, 183–189)

↑, means that the parameter is increased. ↓, means that the parameter is decreased.

Adiponectin acts through its receptors that are broadly expressed in the brain (192), increases insulin sensitivity (193), and promotes fatty acid oxidation and glucose metabolism (194). The hypertrophic adipose cells of obese mice, a seemingly inflammatory environment, synthesize less adiponectin, and the metabolism of fatty acids and glucose is altered. These metabolic alterations persistently observed in the AD brain were suggested to be a stimulus or risk factor for the illness (195). Reduced levels of adiponectin coexist with DM, which increases its relevance (196), whereas the overexpression of adiponectin was shown to prevent the effects of high-fat-diet-induced obesity (183).

Microbial molecules produced in the gut microbiome have also been related to insulin resistance, inflammation and dopamine function (184, 185). Obesity is associated with insulin resistance and an altered microbiome (186). Molecules produced in the gut microbiome, such as lipopolysaccharide (**Table 4**), may induce neuroinflammation, insulin resistance and depressive behavior (186–188). Furthermore, some microorganisms can produce precursors of the neurotransmitter dopamine, which may alter insulin- and dopamine-mediated functions (189).

Under physiological conditions, cannabinoids have neuroprotective and anti-inflammatory effects; they also increase brain glucose uptake by acting through CB2R, suggesting the powerful beneficial effects of these agonists under pathological situations. Additionally, cannabinoid agonists decrease microglial activation, thereby reducing inflammation and cognitive deficits and reducing brain β-amyloid (Aβ)-induced cytokine release (128, 180, 181).

## Therapeutic Approaches

Although many neurodegenerative diseases were initially described several decades ago, an effective therapeutic treatment has yet to be found. AD treatments, based on the amyloid (197) and tau hyperphosphorylation (198) hypothesis, have been used with poor results. Because thiamine deficiency is present in AD patients, drugs targeting altered thiamine metabolism have been also used, producing conflicting results (188). Finally, the use of cocktail therapies, combining drugs acting on multiple pathogenic cascades, has been implemented to treat AD (199).

An important aspect to consider is the therapeutic treatment starting time in the course of the disease. Both AD and T2DM have obvious clinical manifestations 15 to 20 years after the beginning of the pathological entity. This offers a long period of time for starting new therapeutic approaches while the eventual biological targets of the disease are still active.

Among the therapeutic approaches, the use of drugs that modify cerebral metabolic activity is promising. When testing the effects of a new drug on the brain, it is important to check if it can cross the BBB. The noninvasive intranasal route of administration bypasses the BBB to target therapeutics to the brain to treat neurodegenerative disorders such as stroke, AD and PD. Thus, intranasal therapeutics reach the brain directly from the nasal cavity by traveling intracellularly along the olfactory and trigeminal neural pathways (200). Intranasal administration improves brain glucose use and memory in healthy people and in AD patients (201, 202). In addition, when intranasal insulin reaches the brain, it stimulates an enzyme that is also capable of degrading and clearing Aβ from the brain and downregulating GSK**-**3 (166, 167). In addition, insulin protects insulin receptors and synapses from Aβ oligomers, preventing the negative impact of Aβ in neurons (48). Overall, the available evidence indicates that intranasal administration provides an effective supply of insulin to the cerebrospinal fluid compartment with direct access, providing insights into the effects of insulin on the human brain and cognitive function. Many studies support a beneficial effect of insulin on improving cognitive function, although some inconsistent results have been reported. Some possible explanations for the lack of improved cognitive outcomes are (i) the type of intranasal delivery device used, which may differ in efficiency in delivering insulin to the CNS (203); (ii) the type of insulin used; (iii) the type of treatment (acute vs. long-term) when considering the slow onset of insulin-induced memory enhancement; (iv) the CSF-to-plasma insulin ratio in the subjects, which may influence the sensitivity to the cognitive effects of CNS insulin (204); and (v) the cognitive outcomes evaluated.

Today, promising therapeutics include insulin-sensitizing agents such as metformin and PPAR-ϒ agonists, incretin insulin-mimetic molecules, and insulin secretagogues such as glucagon-like peptide-1 (GLP-1) and gastric inhibitory peptide (GIP).

Metformin is a drug broadly used to treat T2DM, improving fasting insulin levels and insulin-dependent hepatic glucose production. Metformin acts differently from insulin by activating AMPK. It was recently reported that metformin can cross the BBB (205). Considering metformin’s insulin-sensitizing properties, it would be reasonable to suggest that metformin may play a beneficial role in dementia, associated with insulin resistance. In this context, an epidemiological study described that metformin treatment reduced the incidence of dementia in diabetic patients (206).

PPAR-ϒ agonists have been used in the treatment of T2DM because they improve insulin sensitivity (207). Additionally, they reduce both Aβ accumulation and neuroinflammation (208). Thus, PPAR-ϒ agonists may improve pathologies related to AD, MCI and T2DM. Treatment with the PPAR-ϒ agonist rosiglitazone was shown not only to reduce fasting insulin levels but also to improve attention and memory in patients during the first stages of AD (209).

GLP-1 stimulates insulin secretion, acting in a glucose-dependent manner. Drugs including gliptins, such as exenatide and liraglutide, mimic the action of GLP-1; other drugs, such as sitagliptin and linagliptin, reduce the degradation of GLP-1, being useful in the therapy of diabetes. GLP-1 readily crosses the BBB, producing several actions through GLP-1 brain receptors. Both exenatide and liraglutide were found to block processes related to neurodegeneration and AD progression in mouse models (210). These findings have been attributed to the neuroprotective properties of those agents (211, 212), reducing Aβ, the neuritic plaque load, and microglial activation (202, 206). Furthermore, GLP-1 mimetics stimulate neurogenesis and improve object recognition and spatial memory, as well as reducing insulin resistance, in MCI and AD (182, 210).

Recently, a further step has been made by the use of multiple receptor agonists to treat diabetes mellitus (213, 214). The dual agonist DA3-CH was more effective than liraglutide in promoting neuroprotection in a mouse model of Parkinson´s disease (213). Thus, dual GLP-1/GIP receptor agonists have better neuroprotective effects than single GLP-1 analogues (214). These results suggest that activating more than one incretin receptor type offers an advantage over using single receptor agonists. In this regard, the effects of triple GLP-1/GIP/glucagon receptor agonists in the APP/PS1 transgenic AD mouse model reduced the total amounts of β-amyloid, neuroinflammation and oxidative stress in the cortex and hippocampus (215). The triple receptor agonist significantly reversed memory deficits, reduced the mitochondrial levels of the proapoptotic signaling molecule BAX, and increased Bcl-2 and BDNF, as well as increasing the levels of synaptophysin and neurogenesis in the dentate gyrus (215). These findings are promising for the design of future AD treatments.

**Figure 3:**
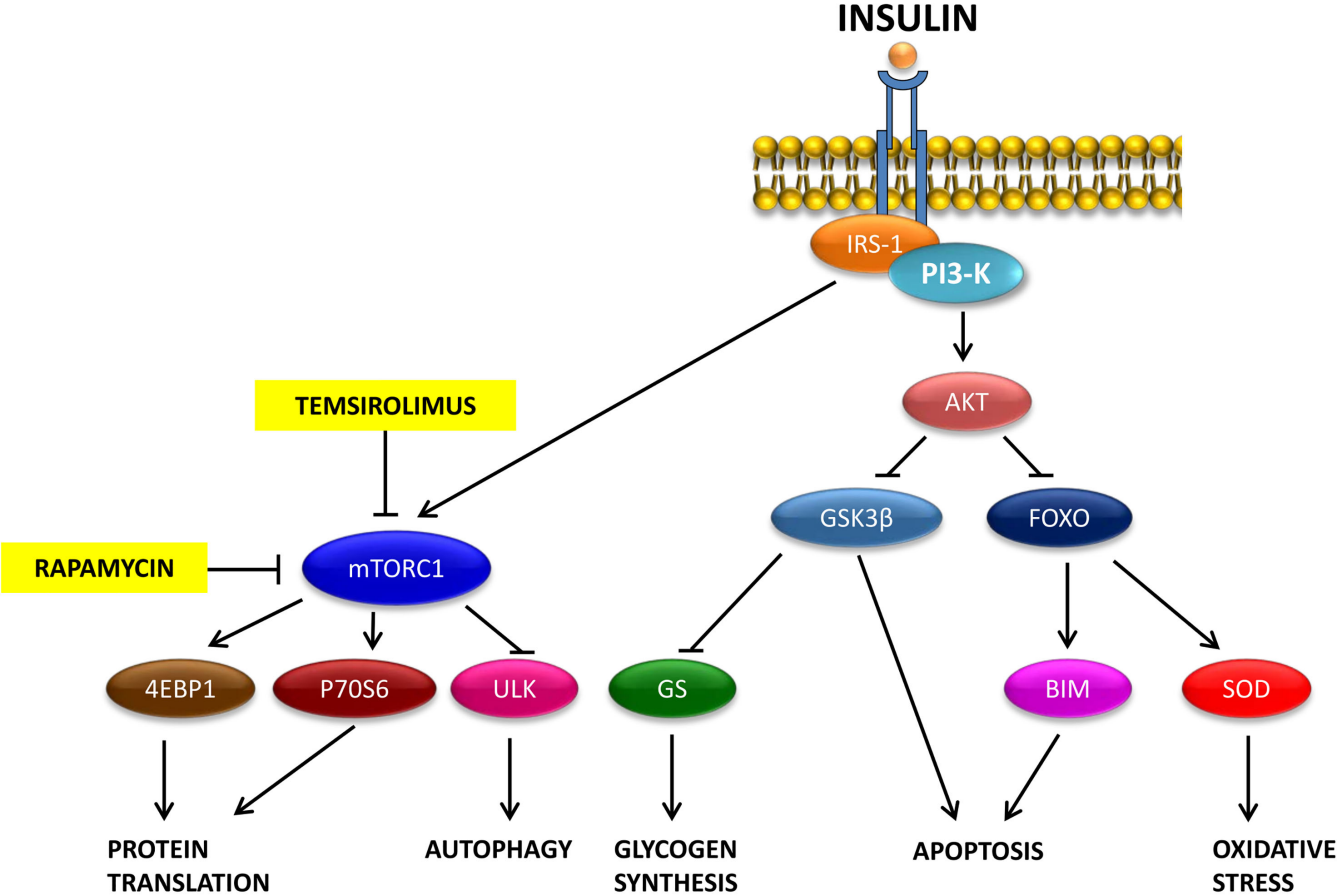
Mechanisms through which rapamycin and temsirolimus prevent the function of the mTORC1 complex.

Target of rapamycin (mTOR) is a serine/threonine kinase that, as a component of the first mTOR complex (mTORC1), promotes cellular growth and limits catabolic processes such as autophagy (**Figure 3**) (216). Therefore, the activation of the PI3K/Akt/mTOR pathway may decrease the autophagic process, facilitating the accumulation of Aβ42. These data support a potential link between Aβ and insulin signaling, which may contribute to insulin resistance.

Although rapamycin prevents the formation of the mTORC1 complex, its potential therapeutic effects are limited because of its low solubility and poor intestinal absorption. Thus, analog molecules such as temsirolimus (**Figure 3**), soluble in water and suitable for intravenous and oral administration, have been developed. Temsirolimus promotes the autophagic clearance of Aβ and improves spatial cognitive functions in an experimental model of AD.

## Conclusions

Until 1978, it was believed that insulin did not play any biological role in the brain. However, since then, a number of studies revealing the presence of high cerebral concentrations of this hormone as much as 25-fold higher than those in the blood circulation, and the presence of insulin receptors in the brain, have opened new frameworks within which to consider the potential central role of insulin. The broad presence of IR suggests a neuromodulatory function for insulin in the brain. Alterations in these functional activities may contribute to the manifestations of several clinical pathologies. Nevertheless, the significance of brain-insulin-mediated effects as reflected by the consequences of restoring, even partially, its altered activity strongly suggests that insulin plays a relevant specific role. These findings encourage the deepening of the research into the mechanisms, signaling pathways, and cause/risk factor nature of these metabolic alterations in the context of neurodegenerative illnesses and future effective therapeutic interventions.

Deficiencies in the major pathways of oxidative and energy metabolism have been reported in patients with neurodegenerative diseases, as extensively discussed throughout this review. Nevertheless, whether those deficiencies are the expression of a single or multiple metabolic alterations needs to be elucidated.

Contributions to brain glucose hypometabolism may be related to glucose transporter alterations, intracellular glucose metabolic abnormalities, changes in thiamine metabolism, and modifications of cerebral metabolic sensors. Reduced GLUT-1 and GLUT-3 expression has mainly been described in the cerebral cortices of AD patients (36–38).

In addition to insulin-mediated pathways, the presence of altered concentrations of thiamine-diphosphate-dependent enzymes (pyruvate dehydrogenase, α-ketoglutarate dehydrogenase and transketolase) in the brains of AD patients supports a role of mitochondrial dysfunction in brain glucose hypometabolism (39, 40).

Lastly, ageing is associated with AD, T2DM and obesity. All these pathologies share several common clinical and biochemical manifestations, such as chronic inflammation, alterations in insulin signaling and insulin resistance, and altered energy metabolism, which contribute to a significant decline in cognitive function with a high risk of dementia. Many patients with profound mental disorders such as schizophrenia and bipolar disorders suffer from metabolic alterations, such as DM and metabolic syndrome (217–220). Comorbidity between mental and metabolic illness may be explained by common risk factors such as stress, which can promote metabolic syndrome and insulin resistance. Accordingly, proinflammatory mediators, elevated concentrations of stress hormones, and insulin resistance favor the expression of mental and metabolic alterations. Interestingly, the medications used to treat metabolic illness have beneficial effects on cognition, such as liraglutide, an agonist of the GLP-1 receptor used to control T2DM (221). In addition, patients with mood disorders have an increased risk of metabolic illnesses, indicating a close relationship between insulin resistance and depression (222). Furthermore, of special relevance is the understanding of insulin resistance, even more so in patients suffering from more than one neurological disorder, such as T2DM and AD patients. Both nosological entities are considered the most prevalent nontransmissible diseases in this century. Accordingly, it is crucial, for scientific, health, economic and social reasons, to gain further insights into brain glucose metabolism and central insulin signaling in neurological disorders.

## Author Contributions

Writing—original draft preparation: EB. Writing—review and editing: FG-O, LG-G, MP, and VH-C. Software: YL-A and VH-C. Conceptualization: MP and JR-A. Supervision: JR-A, EV, LG-G, and FG-O. Funding acquisition: EB and MP. Methodology: MP, LG-G, and EV. Validation: JA, FG-O, and YL-A. Bibliography review: FG-O, LG-G, VH-C, and YL-A. All authors contributed to the article and approved the submitted version.

## Funding

This work was supported by the Ramón Areces Research Foundation, PR2007_18/01, and the Spanish Ministerio de Ciencia e Innovación, Retos PID2019-106968RB-100.

## Conflict of Interest

The authors declare that the research was conducted in the absence of any commercial or financial relationships that could be construed as a potential conflict of interest.

## Publisher’s Note

All claims expressed in this article are solely those of the authors and do not necessarily represent those of their affiliated organizations, or those of the publisher, the editors and the reviewers. Any product that may be evaluated in this article, or claim that may be made by its manufacturer, is not guaranteed or endorsed by the publisher.
